# Silence, depression, and bodily doubt: toward a phenomenology of silence in psychopathology

**DOI:** 10.1080/09515089.2024.2354447

**Published:** 2024-05-20

**Authors:** Dan Degerman

**Affiliations:** Department of Philosophy, University of Bristol, Bristol, UK

**Keywords:** Psychopathology, depression, Merleau-ponty, silence, phenomenology

## Abstract

Despite the relevance of silence in several psychopathologies, first-person perspectives on silence have been largely neglected in the phenomenological scholarship on those conditions. This paper proposes a phenomenological framework for addressing this neglect and demonstrates its usefulness through a case study of empty silence, an experience which can be found in many first-person accounts of depression. The paper begins by surveying research on silence in depression in mental health research and phenomenological psychopathology. Drawing on the thought of Merleau-Ponty, it then outlines a phenomenological framework for explicating the structure of silence experiences in psychopathology. Finally, it applies this framework to articulate the experiential structure and implications of empty silence, with a particular emphasis on the bodily doubt that can flow from repeated experiences of empty silence.

## 1 Introduction

I know exactly when the internal switch in my blood flicked me into depression. [The morning after a busy weekend,] I woke up and knew I had no more running in me. I was in big trouble. I was floating, blood singing in my ears and an unaccustomed silence in the head.

Gwyneth Lewis, *Sunbathing in the Rain* (2003, p. 10)

I felt myself literally slowing down, both in my thinking and in my speech. Sentences took more time than they should have to form; I felt impatient with my own halting words.

Daphne Merkin, *This Close to Happy* (2017, p. 261)

Silence is salient in many aspects of mental illness and healthcare. It is a codified symptom of several mental disorders, including depression (American Psychiatric Association [APA], 2022, p. 187). It has been implicated in the etiology of some mental disorders, again including depression (Jack & Ali, 2011). Moreover, silence is important in common mental healthcare approaches. Some therapists seek to create awkward silences between themselves and their patients to make the latter open up about their problems (Hill et al., 2003). Others consider the patient’s silence as resistance that must be broken down for healing to begin (Lemma, 2016). Still others suggest that tolerance of silence, both one’s own and others, is integral to mental health (Winnicott, 2018).

Despite widespread recognition of the importance of silence in mental illness, first-person perspectives on silence have been largely neglected (Valle, 2019). Mental health researchers and practitioners have tended to approach silence as an objective symptom that can and should be measured independently of patients’ subjective, self-reported symptoms (Peck, 2013). This approach has led some researchers to predict that the silence of patients could soon form the basis for reliable computer-automated diagnoses of depression (Cummins et al., 2015). This approach and its prospects might be appealing for those working within overburdened healthcare systems. However, it may come at a high cost. Not only does it threaten to distort our understanding of silence and its role in mental illness, but it may also exacerbate the marginalization of first-person perspectives on silence in depression and psychopathologies. This could, in turn, compound the suffering and injustices that people with such conditions face.

Meanwhile, scholars of phenomenological psychopathology and philosophy of psychiatry have largely neglected the phenomenon of silence. When silence in and around mental illness does figure in the literature, it is usually as a negative phenomenon in which something that an ill person wants to say is being unjustly suppressed. This is particularly common in the scholarship epistemic injustice in healthcare (e.g., Jackson, 2017; Rosen, 2021). The philosophical tendency to equate silence with suppression and injustice is problematic because it obscures the various meanings and functions that silence can have for people who have been diagnosed with a mental disorder. Saliently, it disregards the possibility that silence may sometimes be a constitutive feature of mental illness, as the experience I focus on in this paper appears to be.

This paper proposes a phenomenological framework for addressing the neglect of first-person experiences of silence and demonstrates its usefulness through a case study of empty silence, an experience that can be found in many autopathographies of depression.

The term silence can, of course, be used to refer to many different phenomena. For one, people often use the term to describe both someone who is not speaking and someone who is speaking about one thing rather than something else. We might say that the former is a literal silence, that is, an absence of words or noise from or in something, while the latter is a metaphorical silence, that is, when speech or noise is being produced but it is covering up something else or it is not understood in the right way. This paper is concerned with literal silences. Literal silence can also be applied to describe disparate phenomena. For instance, it may be used to describe an absence of measurable sound or speech, an absence of audible sound or speech, the ceasing of sound from a particular object in an otherwise noisy environment, or the previous state of an environment that has just been penetrated by salient noise.^1^

In this paper, I am interested specifically in first-person experiences of literal silence and understand silence as a felt absence of speech or noise from or in something.^2^ Though I will focus primarily on speech, it is worth noting that silence, on my definition, does not require a complete absence of perceived sound. Most of us would never claim to have experienced silence if it did. As Don Ihde (2007, p. 81) observes, even in the “ultimate ‘escape’ from noise in the anechoic chamber”, the noise of my body is still with me. And, yet, as we shall see, silence is a ubiquitous, often meaningful, and sometimes painful experience in people’s lives, especially among those living with certain psychopathologies, including depression.

I have chosen to focus on depression in this paper because silence *prima facie* plays a salient role in it, given that silence features in its diagnosis, etiology, therapy, as well as phenomenology. However, silence is a prominent phenomenon in other illnesses and disabilities as well. Notable examples include selective mutism and autism. While I do not consider these in the present paper, the framework I develop here could be productively deployed to explore silence in those and other cases as well.

The paper proceeds as follows. The first two sections survey work on silence in depression in mental health research and phenomenological psychopathology. Through engaging with Maurice Merleau-Ponty’s work on silence and its relationship to speech, the third section outlines a phenomenological framework for explicating the structure of silence experiences in psychopathology. The remaining sections apply this framework to articulate the experiential structure and implications of empty silence, emphasizing the bodily doubt to which repeated experiences of empty silence may give rise.

## 2 Silence in diagnosis and research on depression

In the literature on phenomenological psychopathology and, indeed, the philosophy of psychiatry more broadly, silence in depression is thematized primarily as the outcome of the social silencing of people with that condition. Something rarely recognized in that literature is that silence, in the sense of a person speaking less or not at all, may sometimes be a constitutive feature of the pathology.

As I have sought to indicate with the epigraphs, some people with depression certainly feel as though some silences are part of their pathology. But silence can also be understood as constitutive of depression in a more formal sense; it forms part of the diagnostic criteria for that illness. Both the DSM-5 and the ICD-11 include “psychomotor retardation” as a symptom of a major depressive episode (APA, 2022; World Health Organization [WHO], 2019). This is a broad criterion that can include many different behaviors. The DSM provides some key examples, however, including: “increased pauses before answering”, “speech that is decreased in … amount”, and “muteness”. In the clinical and research literature, these behaviors are often collectively called paucity or poverty of speech. Notably, the DSM specifies that associated phenomena must “not represent merely subjective feelings” of the patient; they must be objectively observable by others (APA, 2022, p. 187).

A related symptom, which also features in both the DSM and the ICD, is “diminished ability to think”, sometimes referred to as thought retardation or poverty of thought. Unlike the previous criterion, this can be based on subjective self-reporting. I mention this symptom here for two reasons. First, I want to suggest that this symptom can correspond to what I will call inner silence. Secondly, some psychiatric textbooks observe that the poverty of thought and speech are connected, which is, of course, very plausible and illustrated by Merkin’s words above. However, they also imply a unidirectionally causal relationship between the two: poverty of thought can precipitate poverty of speech, but not vice versa (e.g., Burton, 2010, p. 23).

It is evidently common for people to think without speaking. But it is equally common for people to speak without thinking; indeed, on the account I provide below, this is assumed to be the norm. That people can speak without thinking may seem a trivial observation. Yet, it suggests that the causal order between the two could go in the other direction: Sometimes, poverty of speech may lead to poverty of thought. Later, I show that a person’s realization that they experience poverty of thought may be preceded by the realization that they experience poverty of speech.

For now, let us return to the psychomotor retardation criterion and poverty of speech. The relevance of poverty of speech in diagnosing depression has been widely recognized in the clinical and research literature (e.g., Peck, 2013; Roose & Devanand, 2013; Whisman, 2017). This is partly because it is considered an objective symptom that can be measured without relying on the subjective self-report of patients. However, the diagnostic relevance of poverty of speech has also been affirmed by a large body of empirical research, which has shown that it is a reliable marker for a major depressive episode (Cummins et al., 2015; Tan et al., 2023; Yamamoto et al., 2020).

Poverty of speech and language changes more broadly in depression are not just relevant to diagnosis. Numerous studies have linked depression to deficits in verbal memory, fluency, and vocabulary (Bora et al., 2012; Douglas & Porter, 2009). One recent meta-analysis suggested that while such verbal impairments are often present from the first diagnosed episode of depression, they tend to be more severe among people who have experienced multiple episodes (Varghese et al., 2022). Importantly, these impairments appear to have a significant negative impact on the lives of people with depression (Rock et al., 2013) and tend to persist even after the affective symptoms of depression have remitted (Semkovska et al., 2019).

Thus, silence is clearly an important phenomenon in depression. Moreover, the research discussed above supports the idea that at least some silences in depression may be part of the pathology itself rather than the consequence of social injustice. Yet, it is notable that mental health researchers have focused on the behavioral aspects of poverty of speech – i.e., whether a person is silent and for how long, their ability to perform some verbal tasks, etc. – without paying much attention to the first-person experiences accompanying poverty of speech (see also Sass & Pienkos, 2015). This paper aims to fill this gap and highlight the need for others to contribute to this project. A few philosophers have already started, as we shall see next.

## 3 Silence, depression and phenomenological psychopathology

While first-person experiences of silence in depression have been largely overlooked, some phenomenologists have begun elucidating related experiences. For example, Louis Sass (1994) has for many years stressed the importance of first-person experiences of language disturbance in schizophrenia, criticizing the neglect of their phenomenology in mental health research (Sass & Pienkos, 2015).^3^ More recently, he and Pienkos (2015) have argued that such experiences may be salient in severe depression as well, which is not surprising since poverty of speech is widely considered a symptom of both depression and schizophrenia (see, e.g., Tan et al., 2023). They link poverty of speech in depression to three types of experiences: an inability to care about expressing oneself, a sense of separation from others, and a sense that one’s pain is ineffable (Sass & Pienkos, 2015, p. 488; see also Ratcliffe, 2015, pp. 2, 202). Each of these experiences could be productively reconceptualized as corresponding to a particular type of silence; in fact, they resonate strongly with some of the silence experiences I have described elsewhere (Degerman, 2023). However, later sections of this paper will show that poverty of speech in depression can involve a broader range of experiences than Sass and Pienkos suggest.

Lucienne Spencer (2023) has recently proposed another experience that can lead to poverty of speech in depression, namely, the experience of hermeneutical injustice. She argues that many experiences of depression that might seem ineffable are, in principle, articulable. But people with depression cannot express those experiences because they lack the hermeneutical resources to do so. They do not have them because we, as a society, have yet to develop and disseminate such resources. Drawing on Merleau-Ponty’s understanding of speech as a part of our habitual body – which I will discuss later – Spencer describes what happens phenomenologically when someone cannot articulate their illness experiences due to hermeneutical injustice. According to Spencer, the illness experience preexists speech expression as an inarticulate “murmur” waiting to be spoken. When the subject of hermeneutical injustice finds themselves in a situation that solicits speech expression about their illness, they pre-reflectively reach for fitting words to articulate the murmur but do not find such words because they do not have them. Hence, they might be silent and the nascent illness experience is held back.

Spencer is right that silence in depression sometimes stems from the contingent absence of adequate language for describing illness experiences. Notably, she also highlights that people with depression are not always as profoundly disconnected from the world as some phenomenological accounts indicate. The world often continues to solicit speech from them, and their inability to respond to such solicitations can be a source of suffering – another theme I will return to later.

While these accounts offer interesting insights, they still leave us with a rather truncated understanding of silence phenomena in depression, particularly what silence is like from the first-person perspective as opposed to what causes people to be silent. Moreover, the experiences that Sass, Pienkos, and Spencer propose as key causes of silence assume that a silent person has something they want to say – even if it is just in the form of a “murmur” – but lacks affective, linguistic, or social resources to express it.^4^ To begin with, that ignores experiences in which the silent person has something they *do not* want to say, though that would still amount to silence holding something back. However, as I will show, silence does not always correspond to an experience of something being held back. Silence can also involve a sense that one *has nothing* to say; this is, for example, the case in what I call empty silence. As we shall see, this experience involves an awareness that one is engaging in neither outward nor inner speech and a feeling that this double-sided silence may be inescapable.

So far, we have seen that silence is an important phenomenon in depression but that first-person experiences of silence have been under-investigated both by mental health researchers and phenomenologists. A current obstacle to filling that gap is that we lack a phenomenological framework for exploring such experiences. In the next section, I will sketch out such a framework.

## 4 Toward a phenomenological framework of silence

In the following two sections, I draw on Merleau-Ponty’s thought to sketch a conceptual framework for exploring experiences of silence. I show that silence experiences in a pre-reflective and transparent form are ubiquitous. I distinguish between three basic kinds of silence experiences: inner silence, outward silence, and outside silence. Finally, I propose that one way these silences can rise to the reflective attention of the subject is through disruptions in what Merleau-Ponty describes as the “near presence of words”.

### 4.1 Merleau-Ponty’s world of silence

Silence is central in Merleau-Ponty’s work (see Kleinberg-Levin, 2008; Mazis, 2016; Toadvine, 2008). While the importance of silence increases in his later writings, it is already prominent in *The Phenomenology of Perception*, which indicates that overlooked silences saturate our lives. A core claim of the book is that many of our experiences are pre-reflective, which is to say – among other things – that they transpire wordlessly and, hence, in a kind of silence. Perception itself, Merleau-Ponty says, communicates with us through a “silent language” (2012, p. 50; see also Walsh, 2017).^5^ When I see my phone, pick it up, unlock it, open an app and begin to scroll, that does not usually involve explicit thoughts like “There is my phone”, “I will pick it up, open an app”, and so on, much less any corresponding speech. My body does all of it silently, without words. But neither does my phone need to speak to me for all those things to happen. My phone is given to me, affords, and solicits it all silently, without speech or sounds. That is not to say that it could not, quite literally, speak to me and tell me to look at it and so on. But it is not necessary.

It is not just in our dealings with inanimate objects that silence is present and, often, goes unnoticed. Merleau-Ponty highlights that pre-reflective silence also forms part of our interactions with other people, even when those interactions involve language. That includes, for example, when we speak.

Across both earlier and later works, Merleau-Ponty argues that our spoken words emerge from silence. In “Indirect language and the voices of silence”, for instance, he writes about “the background of silence which does not cease to surround [speech] and without which it would say nothing” (Merleau-Ponty, 1964a, p. 47). The “background” he has mind here includes the silent appearance of objects discussed above, as well as the silence of things around us that affords us opportunities to speak and be heard (see 1964a, p. 89). We might call this *outside silence* because it refers to a felt absence of speech or noise from things and people outside the subject.

That background of silence also includes an *inner silence* of the speaking subject, that is, the absence of inner speech or noise inside the subject. Merleau-Ponty explains this most clearly in *The Phenomenology of Perception*, where he writes: “The orator does not think prior to speaking, nor even while speaking; his speech is his thought … The ‘thought’ of the orator is empty while he speaks” (185).^6^ What he is pointing to here is that we do not need to articulate each word in our minds before it comes out of our mouths. When someone asks us a question, especially if it is the sort of question we would expect, it solicits our reply without a preceding inner monologue that sets out what we are about to say. We simply begin to articulate an answer out loud, and each word flows from the previous one into the next (see Merleau-Ponty, 2012, p. 180; Merleau-Ponty, 1964a, p. 88). Meanwhile, there is silence in our heads.

Obviously, this is not always the case. For example, a difficult question might lead us to turn inward, engage in inner speech, and begin to articulate an answer in our heads before we speak it. Merleau-Ponty also highlights some illness-related conditions in which speech does *not* come without preceding inner speech (e.g., 2012, p. 180). But often and perhaps generally, inner speech does not precede or overlap with outward speech. Instead, our spoken words tend to appear against a background of inner silence. If that silence is significantly disrupted then our speech may be disrupted, too, as might happen, for example, if we notice that our audience is bored, and that sets off inner speech about how we could stoke their interest.

Inner silence is not only part of the background of our own speech. Merleau-Ponty writes that when we listen to someone speak and we understand what is being said, we do not need to think:

[I]f the expression is successful, we do not have a thought on the margins of the text. The words occupy our entire mind, they come to fulfill our expectation exactly, and we experience the necessity of the speech … but we would not have been capable of predicting it, and we are possessed by it. The end of the speech … will be the lifting of a spell. It is then that thoughts about the speech … will be able to arise. (2012, p. 185)

Engaged listening also involves inner silence. But that silence is fragile, as is perhaps all inner silence. As Merleau-Ponty suggests, if the expression is “unsuccessful” – for example, if the speaker does not express themselves clearly, I am not familiar with the topic, or some noise interferes with my ability to hear the speaker – thoughts often do arise at the margin of the text. The same can occur if the expression is, in a sense, successful. I may, for example, get distracted by inner speech solicited by something particularly interesting that the speaker says. Arguably, however, to the extent that this happens, to the extent that we experience inner speech at the margins of what is being said, we are not quite listening. As Don Ihde (2007) observes, drawing on the same passage as me from *The Phenomenology of Perception*: “there is … is the possibility of only partly hearing what [a speaker] says in the case of in the case of an intrusive inner speech” (159). Inner silence can, thus, be understood as part of the background that facilitates listening.

Precisely because spoken words can occupy us entirely, we often do not notice the inner silence against which we experience someone else’s speech or sounds. If we notice it all, it may well be if the silence is unpleasantly broken, for instance, by something we would rather not think about, or when we realize that we have missed something important that was said because our thoughts distracted us.

Ihde’s remark above draws attention to another type of silence, which is also generally encountered pre-reflectively and which facilitates and constitutes part of the background for listening, namely, my own not-speaking, or what we may call *outward silence*. Without that, I would be literally speaking over the person I could be listening to.

Merleau-Ponty does not explicitly discuss outward silence in its pre-reflective mode. He does sometimes mention the importance of being either pre-reflectively but mainly reflectively silent in certain circumstances for ethical (Merleau-Ponty, 2012, p. 483), epistemic (Merleau-Ponty, 1968, p. 125), or aesthetic reasons (Merleau-Ponty, 1964b, p. 17; see also Mazis, 2016), which is perhaps grounds to assume that he did appreciate the importance of outward silence. However, regardless of whether he did, it seems clear that outward silence is a pervasive and vital experience, particularly in its pre-reflective form. After all, most of the time, no matter how gregarious and extroverted we are, we do not speak.

In sum, I have sought to show in this subsection, firstly, that, on Merleau-Ponty’s account, silence in a pre-reflective form is ubiquitous in objects, other people, and ourselves. The ubiquity of silence on this account has important implications for the framework I am outlining and my argument as it proceeds. If we experience silence all the time without paying much attention to it, then it is probably unlikely that the mere presence of silence determines its phenomenal character. Another key factor in shaping that character – and hence for understanding silence experiences in depression – is how we become aware of silence or, differently put, how we come to encounter silence reflectively.

Along the way, I have also suggested that we can find in Merleau-Ponty’s reflections on silence three different types of silence experiences: (1) outside silence, which is the absence of speech from people and objects around me; (2) inner silence, which is the absence of speech in my mind; and (3) outward silence, which is the absence of speech coming from me.^7^ I want to suggest that we can understand these three as basic silence experiences. We can have much more fine-grained silence experiences, but I think they necessarily involve one or more of these three basic silences. Next, I will draw further on Merleau-Ponty to suggest one reason why these silences tend to remain pre-reflective and transparent and how they sometimes rise to our reflective attention.

### 4.2 Silence in the near presence of language

As noted, Merleau-Ponty thinks we encounter most of the silences I have discussed pre-reflectively, which is to say that we do not notice them; they are, effectively, transparent to us. They form a crucial part of the background of other experiences, but, generally, they do not rise to the foreground. When I speak to you, I do not notice the silence in my head or your silence while you are listening to me, and if you ask me a question, I will not be thinking about my not-speaking.

There are many possible reasons why these and other silences often go unnoticed. I want to consider one reason Merleau-Ponty discusses, namely, what he refers to as “the near presence of the words I know”. Elaborating on what he means by this, he writes: “I relate to the word just as my hand reaches for the place on my body being stung. The word has a certain place in my linguistic world, it is a part of my equipment” (Merleau-Ponty, 2012, p. 186). That is to say, the words we know are part of our habitual body (see also Kee, 2018; Landes, 2013; Spencer, 2023).

What does that mean? On Merleau-Ponty’s account, language is a thoroughly embodied capacity. It is not just that language use requires a body capable of perceiving, recalling, and producing words. We learn to use a word through and with our body, much like we learn to use a tool (424–5). I see or hear someone use a word like “sleet” to describe the hard, cold pebbles presently falling on us from the sky, and I may, thus, grasp the meaning of the word, not unlike I grasp that a hammer has a particular use by seeing someone using it. I might then feel out the word in my mouth or mind, as I might feel out the hammer in my hand if I find one that is not being used. I may then try to use it myself. At first, I might do so while paying careful attention to my pronunciation and how people react in case they think I am misusing it, paralleling my first probing attempts to hit the nail with the hammer. But with repeated use, the word itself becomes just another part of my linguistic world that can be deployed when called for, much like the hammer becomes another part of my toolbox that we reach for when something needs hammering (184–5). Through repeated bodily engagements of this kind, “I come to possess articulatory and sonorous essence as one of the modulations or one of the possible uses of my body” (186). Differently put, our body becomes sedimented with words, which, thereby, obtain a near presence to us.

There are good empirical reasons to think this account reflects something important about our relationship to words (see Kee, 2020). However, we do not need to accept the details of this picture to accept that when someone solicits speech from us, for example, by asking a question, we reach for words intuitively and with a sense of certainty. We expect them to be there, at the tip of the tongue, ready to be spoken, or, somewhere in our head, ready to be thought (see Merleau-Ponty, 2012, p. 168). Moreover, it seems plausible that this sense of certainty is tied to our past experiences of using them with our bodies, e.g., pronouncing, hearing and understanding words in conversation while finding ourselves in certain spaces, feeling a certain way.

This near presence of words explains why we do not ordinarily have to project what we want to say in inner speech before we say it out loud. It also explains why we do not usually notice the inner or, indeed, outward silence that forms part of our interactions with other people. We assume the near presence of the words. Particularly in everyday conversations that do not challenge us, it enables us to slip so seamlessly between seeing, listening, and speaking that we forget, as Merleau-Ponty evocatively puts it, “the thread of silence from which the tissue of speech is woven” (1973, p. 46). Like so many of our everyday experiences, silence recedes to the background and becomes concealed behind that tight web of intentional threads that ties us to the world and our projects in it. To put it more concisely, in everyday life, the near presence of words allows silence to remain transparent.

Merleau-Ponty thought that one of the central tasks of philosophy is to rediscover the silences that language has hidden. In his later works, he became increasingly concerned with how to do so without destroying the character of silence as silence (e.g., Merleau-Ponty, 1968, pp. 170, 212). This is an arcane aim, and Merleau-Ponty scholars have long debated what it might mean (see, e.g., Mazis, 2016; Sallis, 1973; Toadvine, 2008; Wiskus, 2013). Merleau-Ponty himself seems unsure about the answer (see e.g., 1968, pp. 170–171).

However, it is not just through phenomenology or philosophy that silence can rise to the foreground. We routinely notice silence in a reflective mode, even if these instances form just a tiny fraction of the instances in which we encounter silence in a pre-reflective mode.

One familiar situation in which we might become reflectively aware of silence is when a conversation is challenging, for example, if someone asks us an unexpected question to which no answer immediately presents itself. We might say that in such situations, where our sedimented, habitual speech – what Merleau-Ponty calls spoken language – falls short, the near presence of words is disrupted. Effectively, before we know it, we have reached for words but come out empty-handed. In such situations, we might become acutely aware of our outward silence – our not producing a response – and perhaps also of our inner silence if we do not manage to begin to think of an answer. Such a disruption in the near presence of words is not necessarily bad or unpleasant. It can initiate a process of, so to speak, “groping around” for words or some combination of words that could produce a satisfactory answer, a process that characterizes what Merleau-Ponty calls “expressive speech” (1964a, p. 46), which is something along the lines of creative speech and thought.

The previous subsection established that pre-reflective silence is ubiquitous and identified three basic forms of silence, which we can experience pre-reflectively or reflectively. I have summarized these in the table below.^8^

**Table:** 

	Outside silence	Outward silence	Inner silence
*Definition*	An absence of speech or noise around me	An absence of speech or noise coming from me	An absence of inner speech or noise in me
*Pre-reflective*	E.g. Your not-speaking while I am speaking to you	E.g. My not-speaking while I am listening to you intently	E.g. The lack of an inner monologue in me while I am listening to you intently
*Reflective*	E.g. My awareness of your silence as you wait for a response	E.g. My awareness of the lack of words coming from me when I don’t answer your question	E.g. My awareness of the silence inside me when I fail to find an answer a question you’ve asked

This subsection has drawn further on Merleau-Ponty to explain why silence is generally pre-reflective or transparent. That reason is that we ordinarily have a close habitual relationship with words that allows us to draw on them implicitly and, thereby, slip in and out of silence without noticing that it was ever there. This relationship is what Merleau-Ponty refers to as the near presence of words. That near presence can be disrupted and confront us with silence in a reflective mode. Sometimes, the disruption may be severe, amounting to a breakdown in the near presence of words. Merleau-Ponty suggests it can occur in aphasia, for example (2012, p. 180). The next section will show that such a breakdown also characterizes a particular kind of unpleasant silence reported by people with depression, namely, what I call empty silence.

## 5 The empty silence of depression

Across the following two sections, I will apply the framework developed above to analyze an experience of silence that appears in some first-person accounts of accounts of depression.

However, the theoretical detour since the discussion of the research on silence in depression has been long. So, a brief reminder of why we should care about first-person experiences of silence in depression might be useful: Speaking less or not at all and difficulty thinking are both symptoms of a depressive episode, according to the DSM and ICD criteria. A large body of empirical research shows that people who meet the criteria for a depressive episode tend to speak less and that their performance on language tests, particularly those involving the creative use of language, is poorer than that of people without depression. Despite this, little has been written about the phenomenology of silence in depression and only slightly more about the phenomenology of language disruption in depression. Nevertheless, the literature on the latter does provide some insights about the former. These accounts stress two causes of poverty of speech, which in the conceptual framework I have introduced can be understood as a form of outward silence: (1) a lack of appropriate hermeneutical resources to express their feelings or (2) a sense that the world no longer solicits speech from them, that is, a kind of unworlding.

People who have written about their depression often describe experiences of outward silence, but many of those do not seem to be related to either hermeneutical injustice or unworlding. Instead, the experiences they describe appear to involve both a solicitation to speak (or write) and a felt inability to respond, not because they do not have the *right* words but because they do not have *any* words and, hence, nothing to say. This is the experience that I propose to call *empty silence*.^9^ To prefigure the analysis below, it is an experience that involves a solicitation to speak and a breakdown in the near presence of words, which confronts the subject with reflective outward and inner silence. It is an unpleasant experience that, if lived repeatedly, may give rise to bodily doubt in which the subject loses faith in the near presence of words. The epigraphs by Lewis and Merkin at the start are both examples of this but let us have a closer look at another.

The following is an extract from Andrew Solomon’s book *The Noonday Demon* in which he offers several extended reflections on his own experiences with depression. Recalling one severe episode of depression, he writes:

I could not manage to say much; words, with which I have always been intimate, seemed suddenly very elaborate, difficult metaphors the use of which entailed much more energy than I could possibly muster. (Solomon, 2015, p. 51)

Solomon is here clearly describing a kind of outward silence corresponding to poverty of speech. That low energy features in this description could lead us to think that it exemplifies the sort of inability to care about expressing oneself that Sass and Pienkos mention in their analysis of poverty of speech in depression, or the feeling of hopelessness emphasized in phenomenological accounts of depression such as Ratcliffe’s (2015). However, the order of objects in this statement suggests a different experience.

That Solomon’s outward silence becomes an issue for him at all indicates he encountered solicitations to express himself; in this case, it was his father’s presence. Solomon implies that, ordinarily, he would have responded to such solicitations with ease. Back when he was “intimate” with words, he might have been able to do so, like Merleau-Ponty’s orator, without thinking. In depression, those solicitations instead revealed his inability to speak and the “difficult” quality that words had assumed for him. This could be understood as pointing toward a breakdown in the near presence of words, which confronts Solomon with his outward and perhaps also inner silence. Plausibly, it is that experience that raises low energy as a problem for Solomon.

But low energy is considered a core symptom of depression, and Solomon’s account suggests that low energy was a near-constant, debilitating feature of his life. So, you might suggest that even if his relationship with words had changed, the real issue is that he does not have the energy to deal with that change; therefore, his silence can still be reduced to low energy levels – or more phenomenologically charged states or feelings like unworlding or hopelessness. However, if we did, we would miss something important about his experience and others like it. Imagine trying to exit a room through a door, and when you pull the door handle, you realize the door has jammed. The only way out is to force it open, but you are not strong enough. So, you are stuck in the room. Obviously, your general physical weakness is part of what is keeping you there, and more strength would have been enough to get you out. But, under these conditions, your physical weakness has become an issue only because the door has jammed. Knowing that the door is jammed adds something crucial to our understanding of your predicament of being stuck in the room. Similarly, if the near presence of words has been disrupted for people like Solomon in situations where they would ordinarily have spoken, knowing that would add something important to our understanding of their depression.

The above analysis of empty silence and the associated breakdown in the near presence of words resonates with how Karl Jaspers described “classical retardation” in patients with manic depression. In such a state, he writes:

Autonomy over psychic content is limited. The content is not destroyed however as in dementia. *No associations appear; nothing enters consciousness. There is a tendency toward a complete blank*. . . . [O]ften patients are completely mute, and linger for a long time in deep stupor’ (Jaspers, 1963, pp. 211–212; my emphasis).^10^

Both Jaspers’ and my account also cohere with the research literature discussed in Section 2, which suggests that people with depression tend to perform more poorly in creative verbal tasks.

However, Jaspers’ account and that of Sass and Pienkos both fail to highlight something that is palpable in the testimony of Solomon and others who have written about the experience of empty silence. That is the unpleasantness of this type of silence. I would suggest that the unpleasantness of empty silence is inextricably linked to the breakdown in the near presence of words.^11^ After all, neither inner nor outward silence is intrinsically unpleasant, even when experienced reflectively and simultaneously. Occasionally, we all encounter such double-sided silence without doing much more than note it; sometimes, we might even enjoy it. What makes this combination unpleasant in empty silence is that the subject, without the near presence of words, feels trapped in their silence and does not know when or if they will be able to escape it.^12^

## 6 Words in doubt

In this section, I will delve further into the phenomenology of empty silence and argue that it can result in what Havi Carel (2016) refers to as bodily doubt, thereby highlighting one potential and severe consequence of empty silence.

A single experience of empty silence is likely to be brief. To build on Merleau-Ponty’s metaphor, the breakdown in the near presence of words temporarily slackens the tissue of speech to reveal the thread of silence. But once the subject has given up on the effort to respond to the solicitation to speak and other things grab their attention, the tension returns and silence recedes into the background again.

Yet, the impact of repeated empty silence can be profound, leading the subject to doubt their capacity to speak in general. Something like that sort of doubt can be discerned in the following extract from Sally Brampton’s autopathography, *Shoot the Damn Dog*:

What was it I had to say? I can see myself [before depression], sitting at my computer, head bent, writing furiously, hands flying over the keys. [Now, in depression,] I can’t imagine what must have been in my head to make my hands go so fast. (Brampton, 2018, p. 33)

Again, we can see the solicitation to speech expression – in this case, through writing. Finding herself unable to respond to it and confronted with her outward silence, Brampton turned inward. But inside, she seems to have found only more silence and old memories of what she could once do. Whatever enabled her to write – what I am suggesting is the near presence of words – was gone; indeed, she found herself incapable of imagining what it was, suggesting a profound doubt.

We find a reflection on the opposite sort of experience in Brampton’s account of a depressive episode in David Blistein’s (2013) account of recovering from one:

The most important thing is I’m starting to have ideas again. I used to have to carry that little tape recorder because so much was pouring out. Now it’s just a little pocket notebook to jot the occasional one down. It’s a start, an important one…. *I’m much more confident that, when the time comes, the words will be there*. (208; my emphasis)

In this quote, Blistein is celebrating what is, effectively, the return of the near presence of words. But it also suggests that, in depression, he could not be confident that the words would be there when he needed them.

As suggested earlier, the near presence of words – the capacity to draw on language implicitly and, thus, seamlessly move between silence and speech or thought – can be understood as a capacity of the habitual body. We acquire and use words through and with our bodies, not unlike we learn to use tools. Through repeated use, our bodies become sedimented with a vocabulary that we are able to understand, speak, and deploy implicitly and with a sense of certainty. Generally, silence is hidden behind this capacity. In light of this, I would suggest that one could productively speak of the doubt that arises when this capacity becomes unreliable as what Carel calls bodily doubt.

In developing this concept, Carel (2016) begins by describing the relationship that most people have with their bodies in their day-to-day lives as one of pre-reflective bodily certainty. This denotes, Carel says, “the subtle feeling of ‘I can’ that pervades our actions … [a] feeling of possibility, openness, and ability that characterizes routine and familiar actions” (90). Because of this feeling, the body can become the transparent background of our projects. This means, for example, that when I write something, I do not think about my fingers moving, the position of my hands and arms, and so on; I just begin to write. Since Carel explicitly anchors this concept in Merleau-Ponty’s notion of the habitual body, it is unsurprising that it resonates with his account of our relationship to words in habitual situations, as forming parts of our subjective body, which we reach for with a tacit certainty. We implicitly expect them to be available when we want to say something, much like we expect our fingers to be there when we start typing on the keyboard. The near presence of words can, hence, also be understood as involving pre-reflective bodily certainty and a feeling of “I can”; this allows our silences to become the transparent background of other experiences. As Merleau-Ponty puts it, when we use habitual expressions, “the gaps and element of silence are obliterated” (1973, p. 46).

By contrast, bodily doubt involves the loss of that feeling of “I can” and its replacement by a feeling of “I cannot”. This general feeling of inability can be directed at a particular aspect of bodily functioning or be generalized to overall functioning. Beyond that feeling, Carel identifies three key features of bodily doubt. The first is a *loss of continuity*, in which implicit abilities no longer function as they used to, forcing the subject to renegotiate their participation in the shared world (98). The second is *loss of transparency*, in which an aspect of bodily functioning that the subject used to take for granted pre-reflectively becomes the object of pervasive reflective attention in situations where they would previously have been mobilized implicitly. The final feature is a *loss of faith* in one’s body. This refers to the disruption of those implicit beliefs about the subject’s body that allows them to go about their day-to-day lives and pursue specific projects.

These features of bodily doubt are all discernible in the accounts of empty silence discussed above. All the autopathographers I have quoted testify to the loss of continuity that the experience involved for them. For example, Solomon describes the collapse of his lifelong “intimacy” with words, and Lewis recounts the inner silence that replaced the inner speech to which she was accustomed. Empty silence confronted each with their inability to participate in the world as they used to do, that is, easily responding to solicitations to speak and write, and many of them describe how they began to avoid situations in which they would be solicited to do so.

The loss of transparency that may flow from empty silence comes through particularly clearly in Brampton’s account. We can see that, for her, two closely related aspects of bodily functioning lost their transparency through empty silence: firstly, the near presence of words – she forlornly recalled her “hands flying over the keys” – and, secondly, her own silence – not only was she unable to write but she could not even find anything in her head about which to write. Similarly, Blistein’s relief that words would once again be there when needed after he had recovered points toward an experience of having felt that, where there used to be words, there was instead silence. Hence, empty silence may simultaneously render both the near presence and the silence that prevails when words are not there pervasively opaque.

The opacity of silence is perhaps the most noteworthy and significant aspect of the bodily doubt engendered by repeated experiences of empty silence. Recall that the Merleau-Pontian assumption is that pre-reflective, transparent silence, including our own inner and outward silence, is ubiquitous and forms an implicit part of the background of countless activities. In other words, there is a lot of silence that could be attended to. If, through repeated experiences of empty silence, a person’s inner and outward silences become opaque and a sign of inability, then the sense of bodily doubt might become inescapable. After all, as noted earlier, most of us are silent most of the time.

Finally, while a loss of faith might be inferred from all three accounts, with each person finding they could no longer use words as before, it is perhaps most salient in Blistein’s. The certainty that words would again be there when he needed them defined his sense of recovery. This indicates that the loss of faith in the near presence of words affected him profoundly. Repeated experiences of empty silence can prompt such loss of faith by sensitizing a person to solicitations to speak and their silence in the face of those solicitations. For example, we can imagine Blistein sitting around the dinner table listening to his family discuss their day, seeing his computer in the corner, or having a friend visit. In each situation, he might have had the sense or thought about how he would ordinarily throw himself into these situations in speech or writing and realized that he now was not, that he was outwardly silent. He might have turned inward, racking his brain to see if it would turn something out, but only finding more silence. Hence, each experience of empty silence would have confirmed that he was, in fact, unable to rely on the near presence of words as he used to. This may entrench a persistent sense of “I cannot” from which it can be difficult to recover fully, a difficulty that is potentially reflected in the research showing that language impairments often persist even after the affective symptoms of a depressive episode have remitted (Semkovska et al., 2019).

Empty silence can, thus, engender a sense of bodily doubt centered on a person’s sense that they cannot rely on the near presence of words to break their silence. That doubt, I have suggested, may be intensified ubiquity of silences that may become experienced as opaque and inescapable. As the first-person accounts quoted show, this doubt may significantly compound the suffering of the person with depression.

## 7 Conclusion

My aim in this paper has been to demonstrate the importance of investigating the phenomenology of silence in psychopathology and show that such investigation can yield important insights about those conditions. I have done so through a case study of silence in depression.

I began by surveying existing work on silence in depression, demonstrating that while silence is a clinically significant phenomenon in depression, little work has been done to elucidate the phenomenology of such experience. To provide a resource in filling that gap, I sketched out a phenomenological framework inspired by the work of Merleau-Ponty. Central to this framework is the distinction between three basic kinds of silence experiences – inner, outward, and outside silence – each of which can be given pre-reflectively or reflectively. In their pre-reflective form, these silences are taken to be ubiquitous, forming an implicit background to countless activities. I proposed that one instance in which silence rises to reflective awareness is when our ordinary habitual relationship with language, which allows us to draw on words implicitly – what Merleau-Ponty calls the near presence of words – is disrupted. I then applied this to analyze first-person accounts of a kind of silence experience in depression that I termed empty silence. This is an unpleasant experience that involves a solicitation to speak and a breakdown in the near presence of words that confronts the subject with reflective outward and inner silence. I proceeded to argue that if lived repeatedly, it may give rise to a kind of bodily doubt in which the subject no longer feels they can rely on the near presence of words and the silences that surround them become opaque and, seemingly, inescapable.

My analysis has left many loose ends for myself and, hopefully, others to pursue. One way it could be extended is by considering the relationship between empty silence and rumination. Closely linked to depression both phenomenologically and causally, rumination is a kind of intrusive negative inner speech that arises and continues, although the subject might want it to stop (Nolen-Hoeksema et al., 2008). *Prima facie*, it might sound like rumination is the opposite of empty speech. Yet, there are experiential similarities between them, as both are, for example, unpleasant. Both also seem to involve a breakdown in the near presence of words, though, in empty silence, they will not come, and, in rumination, they will not leave. Something along these lines is suggested by Lewis’s reflections on her experience of depression: “[T]he commentary box in my head seemed to have a faulty connection. It had two modes: screaming and radio silence” (2003, p. 27).

Other empirical evidence also hints at a connection between empty silence and rumination, suggesting that the poor performance of people with depression on creative verbal tasks can be at least partly explained by ruminative thoughts and tendencies interfering with memory and concentration (Joorman, 2009, p. 305). It is possible that experiences of rumination and empty silence can form a kind of self-reinforcing symbiosis; ruminative thoughts lead a person to be ill-prepared to participate in conversations that do not relate to those thoughts, which might confront them with empty silence, which leads to more rumination, and so on.

A related strand worth pursuing is the relationship between empty silence and peaceful silence in depression. Peaceful silence *prima facie* shares core experiential features with empty silence. Yet, unlike empty silence, peaceful silence is pleasant. Some people with depression who are plagued by rumination actively pursue it, and there are therapies to help them achieve it. I suspect that the phenomenological framework sketched above, particularly the notion of the near presence of words, could help us understand what makes those two silence experiences so different.

While both the avenues for further investigation I have outlined here pertain to depression and psychopathologies that involve depressive episodes, phenomenological studies of silence could be fruitful in the context of other psychopathologies as well. I hope the phenomenological framework I have outlined here will aid such investigations.

## Notes

1 I will regularly refer to objects as silent in this paper. As an anonymous reviewer of this paper observed, such talk may be confounding since, arguably, we do not expect objects to talk. However, there are several reasons why speaking of objects as silent is justified and important to the broader project of elucidating the experiences of silence in mental illness. Firstly, it is consistent with Merleau-Ponty’s analysis of silence, which draws attention to that things generally appear to us non-linguistically. Secondly, while most objects do not talk, we increasingly encounter objects capable of producing speech (e.g., smart speakers, etc.) and are surrounded by objects capable of making (or being made to produce) noise. The lack of noise from such objects can constitute salient silence experience for anyone; think, for example, of a squeaky door hinge that has been bothering you but that becomes silent after you have oiled it. But the silence of objects may be a particularly salient experience in some mental illnesses and disabilities that involve a sensitivity to noise, such as autism. Finally, in some mental illnesses, such as schizophrenia and others that involve hallucinations, objects may be capable of producing speech much like a person. Hence, talk of silent objects in developing the framework of this paper may make it more versatile and applicable to other psychopathologies, illnesses, and disabilities.

2 The content of silence experiences is subject to debate (see, e.g., Gow, 2021; Skrzypulec, 2022).

3 Language disturbance figures centrally in the phenomenologically inspired interview format “Examination of Anomalous World Experience” (EAWE), lead-authored by Sass (see Sass et al., 2017). However, this instrument was primarily developed to explore experiences related to schizophrenia spectrum conditions. While its authors acknowledge that it might have broader uses, it has apparently not yet been applied to explore language disturbance in depression.

4 Sass and Pienkos do mention a fourth potential cause of poverty of speech but pass over it rather quickly: “an overpowering sense of nothingness or indistinctness [that] may create the feeling that there is, in fact, nothing to describe” (2015, p. 484). This resembles what I call empty silence and may well be an instance of it.

5 Ted Toadvine (2008) observes that Merleau-Ponty’s idea that perceptual experience and perhaps experience in general appears to be based on Husserl description of experience in *Cartesian Meditations* as “dumb” (Husserl, 1999, pp. 38–39), or in Merleau-Ponty’s French translation as “muette” meaning “mute” (Merleau-Ponty, 2012, p. 228; see also 1968, p. 129).

6 While I concentrate on thought in the form of inner speech in this paper, neither I nor Merleau-Ponty think that all unexpressed thought transpires as inner speech. Visual thinking, for one, features regularly *The Phenomenology of Perception* (e.g., Merleau-Ponty, 2012, pp. 405–6, 411, 453).

7 Other types of silence can be discerned in Merleau-Ponty’s work as well. He also discusses silences that could be understood, using Alessandra Tanesini’s (2018) vocabulary, as informative or eloquent (e.g., Merleau-Ponty, 1964a, p. 76), in which silence conveys a specific and possibly intentional meaning. He also describes what Bernard Dauenhauer (1980) calls “in-between silences” and “fore-and-after silences”, which are the moment in, before and after utterances that make them intelligible to listeners (e.g., Merleau-Ponty, 1964a, p. 45). These are not the sorts of silence I am interested in here, though it seems these too could be articulated in terms of the three basic silence experiences I describe.

8 The case study that follows focuses on an experience involving inner and outward silence. Hence, outside silence is not subject to further investigation in this paper. Nevertheless, experiences of outside silence are doubtlessly an important phenomenon in some mental illnesses. Those experiences warrant further investigation, which the proposed framework can facilitate.

9 In a previous paper, I called this “depressed silence” (Degerman, 2023), but I now believe the term empty silence better captures the nature of the experience.

10 Interestingly, in *Philosophy*, Vol. II, Jaspers mentions in passing a similar type of experience that he too calls “empty silence, the stillness in which I express nothing because I feel nothing” (1970, p. 68).

11 The unpleasantness of such experiences, not to mention the functional difficulties that they can produce, might be part of why people with depression often complain about cognitive impairment (Beblo et al., 2023).

12 Sarah Maitland (2009, p. 92) offers a similar analysis of what makes the difference between pleasant and unpleasant silence, suggesting that it has to do with whether it is chosen.
